# Identification of a novel SEREX antigen family, ECSA, in esophageal squamous cell carcinoma

**DOI:** 10.1186/1477-5956-9-31

**Published:** 2011-06-23

**Authors:** Akiko Kagaya, Hideaki Shimada, Tooru Shiratori, Mari Kuboshima, Kazue Nakashima-Fujita, Mari Yasuraoka, Takanori Nishimori, Shunsuke Kurei, Takahisa Hachiya, Akihiro Murakami, Yutaka Tamura, Fumio Nomura, Takenori Ochiai, Hisahiro Matsubara, Masaki Takiguchi, Takaki Hiwasa

**Affiliations:** 1 Department of Biochemistry and Genetics, Chiba University, Graduate School of Medicine, Chuo-ku, Chiba 260-8670, Japan; 2 Department of Frontier Surgery, Chiba University, Graduate School of Medicine, Chuo-ku, Chiba 260-8670, Japan; 3 Department of Surgery, School of Medicine, Toho University, Ota-ku, Tokyo 143-8541, Japan; 4 Product Development Department, Medical & Biological Laboratories Co., Ltd., Ina, Nagano 396-0002, Japan; 5 Department of Bioinfomatics, Chiba University, Graduate School of Medicine, Chuo-ku, Chiba 260-8670, Japan; 6 Department of Molecular Diagnosis, Chiba University, Graduate School of Medicine, Chuo-ku, Chiba 260-8670, Japan; 7 Gastroenterological Center, San-Ai Memorial Hospital, Chuo-ku, Chiba 260-0806, Japan

## Abstract

**Background:**

Diagnosis of esophageal squamous cell carcinoma (SCC) may improve with early diagnosis. Currently it is difficult to diagnose SCC in the early stage because there is a limited number of tumor markers available.

**Results:**

Fifty-two esophageal SCC SEREX antigens were identified by SEREX (serological identification of antigens by recombinant cDNA expression cloning) using a cDNA phage library and sera of patients with esophageal SCC. Sequence analysis revealed that three of these antigens were similar in amino acid sequences, and they were designated as ECSA (esophageal carcinoma SEREX antigen)-1, -2 and -3. The ECSA family was also similar to an EST clone, hepatocellular carcinoma-associated antigen 25a (HCA25a). Serum antibody levels to ECSA-1, -2 and -3 were significantly higher in patients with esophageal SCC than in healthy donors. Based on the conserved amino acid sequences, three peptides were synthesized and used for enzyme-linked immunosorbent assays (ELISA). The serum antibody levels against one of these peptides were significantly higher in patients with esophageal SCC. This peptide sequence was also conserved in FAM119A, GOSR1 and BBS5, suggesting that these are also ECSA family members. Reverse transcription followed by quantitative PCR analysis showed that the mRNA expression levels of ECSA-1, -2 and -3 and FAM119A but not of HCA25a, GOSR1 and BBS5 were frequently elevated in esophageal SCC tissues.

**Conclusions:**

We have identified a new gene family designated ECSA. Serum antibodies against the conserved domain of the ECSA family may be a promising tumor marker for esophageal SCC.

## Background

Esophageal squamous cell carcinoma (SCC) is one of the most malignant tumors. The 5-year survival rate varies between 20% and 40% [1-3]. Despite improvements in surgical techniques and adjuvant chemoradiotherapy, many patients suffer from rapid recurrence of the disease and have a poor prognosis [4]. Because these tumors are aggressive, patients often present with systemic involvement due to delayed diagnosis [3]. Although several tumor markers have been identified in esophageal SCC, they are not sufficiently prevalent to allow for use as a general diagnostic tool [5-7].

It has been known for several decades that the immune system is able to recognize tumor cells [8,9], and by testing for autoimmunity against tumor-associated antigens it is possible to identify tumor markers. This analytical method developed by Sahin *et al*. is called SEREX (serological identification of antigens by recombinant cDNA expression cloning) [10]; it involves immunoscreening with autologous or allogeneic sera of expressed cDNA libraries prepared from tumor specimens. Since the antigens are easily identified by sequencing the isolated cDNA clones, SEREX is suitable for large-scale screening of tumor antigens. SEREX has been applied to a variety of human tumor types and has successfully identified over 1,000 novel tumor antigens [11].

The SEREX method has been applied to esophageal SCC and has also identified NY-ESO-1, a gene product of testicular cells. This antigen is overexpressed in various types of cancer cells [12]. SEREX analysis has also led to the isolation of several antigens known to be associated with malignancy, including a mutated version of p53 tumor suppressor protein in esophageal cancer [13,14]. The presence of serum p53 antibodies has been associated with poor prognosis [15].

In the previous series of SEREX screening in esophageal SCC, we identified several new SEREX antigens, including TROP2, SURF1, SLC2A1, TRIM21, myomegalin and makorin 1 [16-25]. In the present series, we have identified a novel gene family, designated ECSA (Esophageal Carcinoma SEREX Antigen), by SEREX screening and have evaluated the clinicopathological significance of serum ECSA antibodies (s-ECSA-Abs) in patients with esophageal SCC.

## Results

### Serological screening of cDNA Library

**Figure 1:**
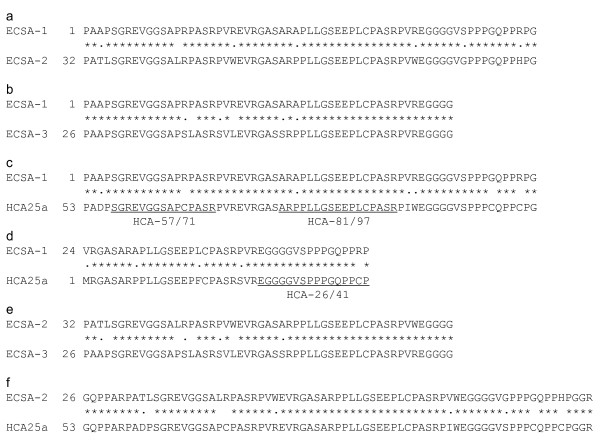
**Amino acid sequence homology among ECSA-1, -2 and -3 and HCA25a**. Identical amino acids and homologous amino acids are indicated by asterisks and dots, respectively. The position numbers of each first amino acid are indicated. The positions of the synthetic peptides, HCA-57/71, HCA-81/97 and HCA-26/41, are also shown.

A phage expression library was constructed from the mRNA of an esophageal SCC cell line, T.Tn. A total of 3 × 10^6 ^clones of cDNA were screened using sera from three patients with esophageal SCC, and 52 reactive clones were isolated. DNA sequence analysis and homology search, using the National Center for Biotechnology Information (NCBI) databases, revealed that the DNA sequences of three clones, 10Q3-1, 12N1-1 and 12O1-1, were similar but not identical. Almost identical sequences of 10Q3-1, 12N1-1 and 12O1-1 were found on chromosomes X, 9 and 19, respectively, suggesting that these three clones were independent. Thus, these genes belonged to a novel gene family, designated as the ECSA (esophageal carcinoma SEREX antigen) family. Clones 10Q3-1, 12N1-1 and 12O1-1 were designated as ECSA-1, -2 and -3, respectively. These genes were also similar to an EST clone, hepatocellular carcinoma-associated antigen (HCA25a) (Accession number: AF469043). The coding sequences of ECSA-1, -2 and -3 are not known yet; their putative amino acid sequences are shown in Additional File 1. Nucleotide and amino acid sequence homologies among these genes are shown in Tables 1 and 2. ECSA-1 and HCA25a were 91.6% homologous in the nucleotide sequences and 86.8% homologous in the amino acid sequences. The amino-terminal region of ECSA-1 was homologous not only to HCA25a amino acids 53 through 117 but also to HCA25a amino acids 2 through 41 (Figure 1), indicating that HCA25a contains internal repetitive sequences. ECSA family sequences are highly conserved, especially in repetitive regions.

**Table 1:** Nucleotide sequence homology among ECSA family members and HCA25a.

	ECSA-1	ECSA-2	ECSA-3	HCA25a
ECSA-1	-	87.0	70.0	91.6
ECSA-2	87.0	-	79.3	84.8
ECSA-3	70.0	79.3	-	73.0
HCA25a	91.6	84.8	73.0	-

Percent homologies of nucleotide sequences are shown.

**Table 2:** Amino acid sequence homology among ECSA family members and HCA25a.

	ECSA-1	ECSA-2	ECSA-3	HCA25a
ECSA-1	-	89.4	87.5	86.8
ECSA-2	89.4	-	85.4	80.0
ECSA-3	87.5	85.4	-	83.3
HCA25a	86.8	80.0	83.3	-

Percent homologies of amino acid sequences are shown.

### Presence of serum ECSA antibodies (s-ECSA-Abs) in patients with esophageal SCC

**Figure 2:**
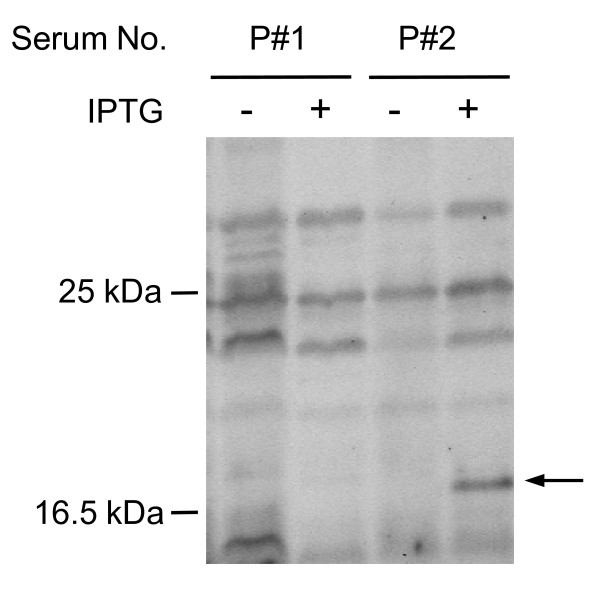
**Recognition of ECSA-2 by serum antibodies in patients with esophageal SCC**. *E. coli *containing ECSA-2 cDNA expression plasmids was incubated with (+) or without (-) IPTG for 2.5 h, with cell lysates subsequently subjected to Western blot analysis using sera from patient-1 (P#1) and patient-2 (P#2) with esophageal SCC. An arrow indicates the IPTG-induced polypeptide that represents the ECSA-2 cDNA product.

To confirm the presence of s-ECSA-Abs in patients with esophageal SCC, Western blotting was performed using bacterially expressed ECSA gene products as described previously [16-19]. Figure 2 shows representative positive and negative results for s-ECSA-2-Abs. Only signals that appeared in the samples pretreated with IPTG were judged as sero-positive. The size of the IPTG-dependent polypeptide was approximately 15 kDa, which was consistent with the estimated size of the fusion protein of pBluescript II-encoded β-Gal and the amino-terminal truncated ECSA-2 protein. The results shown in Figure 1 indicate that the serum of patient-1 (P#1) was sero-negative for s-ECSA-2-Abs, while the serum of patient-2 (P#2) was sero-positive. We examined the sero-positivity for s-ECSA-2-Abs in sera from 40 patients with esophageal SCC and 20 healthy donors. Positive reactions were detected in sera of 40% (16 of 40) of patients, whereas the sero-positivity for s-ECSA-2-Abs in healthy donors was only 10% (2 of 20).

### ELISA assay to detect serum antibodies

**Figure 3:**
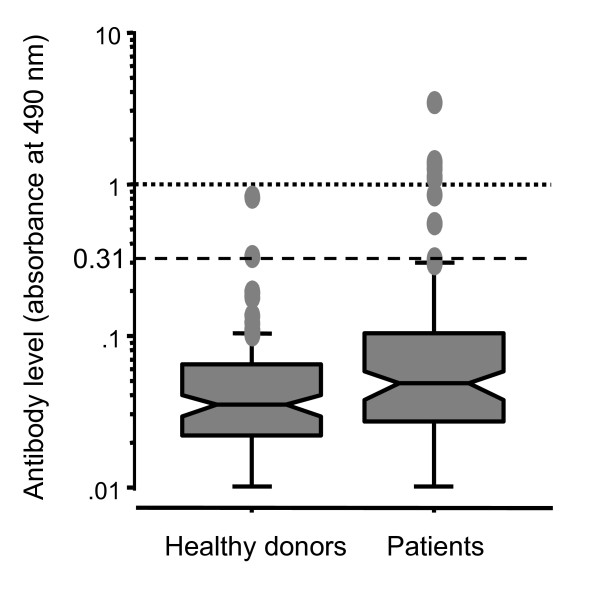
**s-ECSA-2-Ab levels examined by ELISA**. The levels of s-ECSA-2-Ab were measured by ELISA using purified GST-ECSA-2 protein and control GST protein. The box plots display the 25th, 50th, and 75th percentiles. The upper and lower cross bars of the box represent 90th and 10th percentiles, respectively. The position of a cut-off value (Average + 3 SD; 0.31) is shown. Values higher than 90th percentile are shown by dots.

Although it is possible to evaluate the presence or absence of serum antibodies by Western blotting, only a limited quantitative estimation is possible. ELISA assays were performed on sera from patients and healthy donors using a recombinant antigen protein to analyze the levels of s-ECSA-Abs quantitatively. cDNA of ECSA-2 was recombined into pGEX-4T, which produced the GST-ECSA-2 fusion protein after treatment with IPTG. The fusion protein was affinity-purified by glutathione-Sepharose (Additional File 2). The levels of serum antibody in patients with esophageal SCC were significantly higher than those in healthy donors (0.145 ± 0.483 versus 0.050 ± 0.087, P = 0.0026) (Figure 3). The levels of s-ECSA-2-Ab were divided into two groups: A serum level less than 0.31 was considered normal, as this was the mean + three standard deviations (SD) of s-ECSA-2-Ab level of healthy donors; abnormal or positive values were higher than 0.31. The positive rate of patients with esophageal SCC was higher than that of healthy donors (15.3% versus 1.0%). ELISA using purified recombinant ECSA-1 and -3 proteins revealed that serum antibody levels against ECSA-1 and -3 also were significantly higher in patients with esophageal SCC than in healthy donors (Table 3).

**Table 3:** Serum antibody levels against ECSA proteins or peptides examined by ELISA.

	ECSA-1		ECSA-2		ECSA-3		HCA-26/41		HCA-57/71		HCA-81/97		HCA-74/88		HCA-85/97		HCA-88/97	
									
	HD	P	HD	P	HD	P	HD	P	HD	P	HD	P	HD	P	HD	P	HD	P
Average	0.004	0.132	0.050	0.145	0.011	0.230	0.009	0.010	0.025	0.067	-0.018	0.086	0.024	0.023	-0.012	-0.016	0.032	0.021
SD	0.030	0.387	0.087	0.483	0.087	0.656	0.037	0.015	0.071	0.160	0.061	0.189	0.015	0.013	0.061	0.105	0.031	0.043
Positive rate	2/118	31/146	1/98	11/72	1/74	11/68	1/42	2/63	1/42	6/63	0/42	14/63	1/42	0/42	0/42	1/42	0/42	0/42
(%)	1.7	21.2	1.0	15.3	1.4	16.2	2.4	3.2	2.3	9.5	0.0	22.2	2.4	0.0	0.0	2.4	0.0	0.0
P value (T-test)	0.0001		0.0026		0.0079		0.8848		0.0702		0.0001		0.6690		0.8210		0.1738	

Synthetic peptides used were; HCA-26/41, biotin-EGGGGVSPPPGQPPCP; HCA-57/71, biotin-SGREVGGSAPCPASR; HCA-81/97, biotin-ARPPLLGSEEPLCPASR; HCA-74/88, biotin-REVRGASARPPLLGS; HCA-85/97, biotin-PLLGSEEPLCPASR; and HCA-88/97, biotin-SEEPLCPASR. ECSA-1, 2 and 3 were GST-fusion proteins.

Next, we evaluated the relationship between clinicopathological parameters and the presence of s-ECSA-Abs. The survival of s-ECSA-Ab-negative patients was superior to that of s-ECSA-Ab-positive patients, although the differences were not statistically significant (P = 0.1851, Figure 4). There was no correlation between the presence of s-ECSA-Abs and other clinicopathological variables such as gender, age, tumor location, tumor depth, N factor or M factor (Table 4). A multivariate analysis was also performed to evaluate the prognostic significance of s-ECSA-Abs. Although TNM factors were independent risk factors for survival, s-ECSA-Abs were not (data not shown).

**Figure 4:**
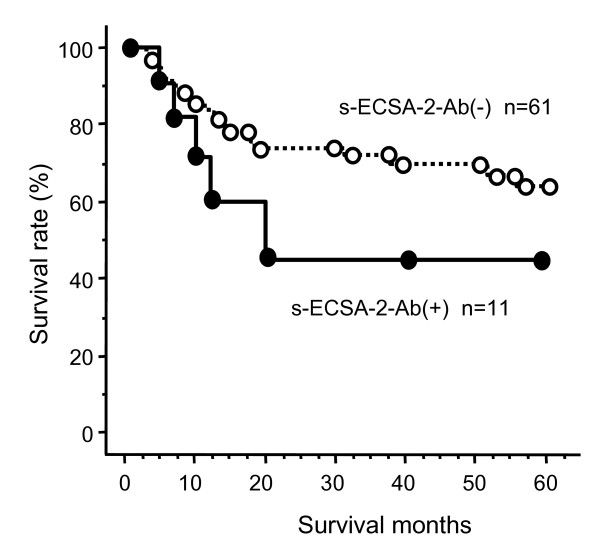
**Kaplan-Meier overall survival curves for the patients with and without s-ECSA-2-Abs**. The P value calculated by the log-rank test was 0.1851.

**Table 4:** Relationship between s-ECSA-2-Abs and the clinicopathological variables in patients with esophageal SCC.

Variables (No. of patients)		No. of patients with s-ECSA-2-Abs (positive rate %)	P-value
Gender	Male (63)	10 (16)	>0.9999
	Female (9)	1 (11)	
			
Age	<65 years (31)	6 (19)	0.5132
	≥65 years (41)	5 (12)	
			
Tumor location	Upper (13)	1 (8)	0.6757
	Lower (59)	10 (17)	
			
Tumor size	<50 mm (39)	4 (10)	0.3245
	≥50 mm (33)	7 (21)	
			
Tumor depth	T1, T2 (35)	3 (9)	0.1906
	T3, T4 (37)	8 (22)	
			
N factor	N0 (25)	1 (4)	0.0836
	N1 (47)	10 (21)	
			
M factor	M0 (61)	8 (13)	0.3562
	M1 (11)	3 (27)	
			
Stage	I, II (37)	3 (8)	0.1073
	III, IV (35)	8 (35)	

### ELISA assay using synthetic peptides

**Figure 5:**
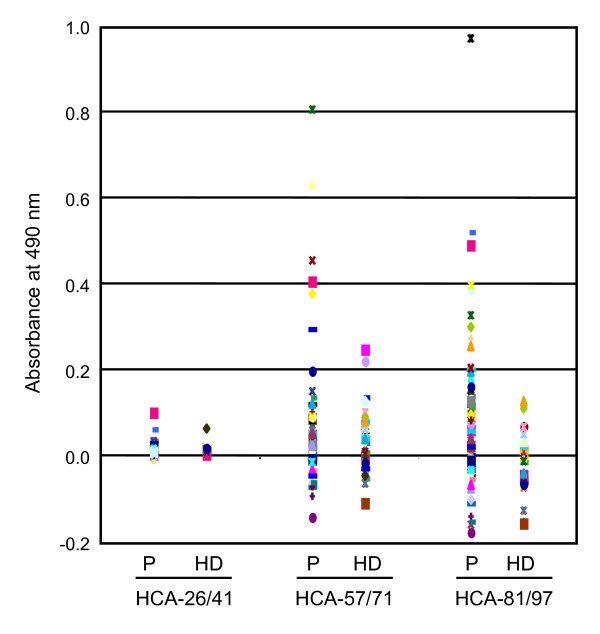
**Serum antibody levels against the synthetic peptides, HCA-26/41, HCA-57/71 and HCA-81/97**. The levels of serum antibodies against synthetic peptides in sera of patients with esophageal SCC (P) and healthy donors (HD) were examined by sandwich ELISA.

The isolated ECSA clones may not be full-length because they did not contain an initiation codon in the 5'-terminal region, and the putative open reading frame is short (Additional File 1). However, the results of Western blotting (Figure 2) and ELISA (Figure 3) suggest that the epitope is located in the short and conserved region of the ECSA family peptides. Thus, we synthesized short peptides based on the repetitive sequences of HCA25a, and used them as antigens to examine the levels of serum antibodies and to characterize the peptide epitope. Three peptides, HCA-26/41, HCA-57/71 and HCA-81/97 were designed and used for ELISA (Figure 1). The serum antibody levels against HCA-81/97 were significantly higher in patients with esophageal SCC than in healthy donors (Figure 5, Table 3). Similar results were also observed against HCA-57/71, but the difference between patients and healthy donors was insignificant. The antibody levels against HCA-26/41 were very low in both patients and healthy donors. When the cut-off value was defined as the average + 3 SD, 22% of patients with esophageal SCC were positive for the serum anti-HCA-81/97 antibody but none of the healthy donors were positive (Table 3). To define the epitope site more precisely, the antibody response to other synthetic peptides, such as HCA-74/88, HCA-85/97 and HCA-88/97, was examined by ELISA. None of these peptides showed significant differences in the serum antibody levels between patients and healthy donors (Table 3), suggesting that an almost full-length sequence of HCA-81/97 is required to react with the serum antibodies.

### Identification of other ECSA family members

**Figure 6:**
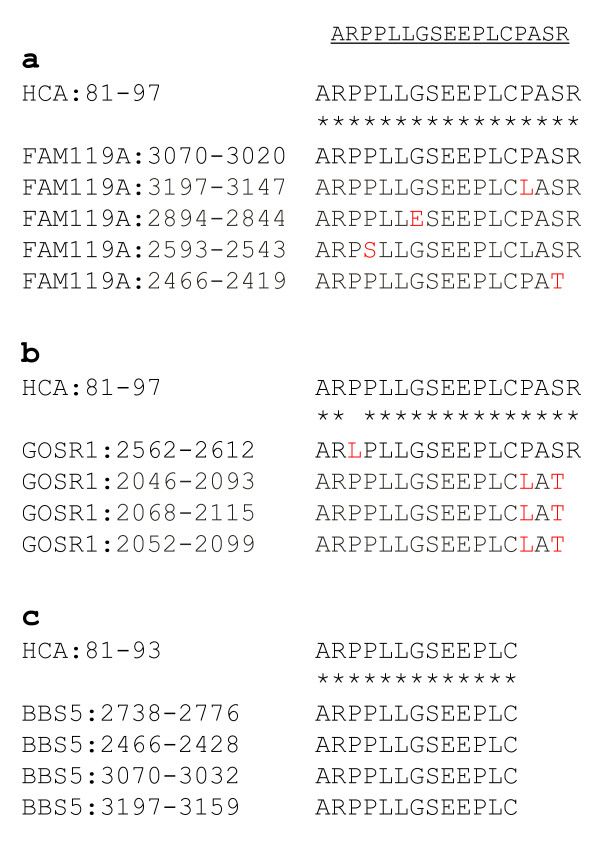
**Amino acid sequence homology between HCA-81/97 and FAM119A, GOSR1 and BBS4**. The sequences of FAM119A (a), GOSR1 (b) and BBS4 (c) are deduced amino acid sequences from the nucleotide sequences of cDNA, of which the position numbers are shown. Non-identical amino acids are shown in red.

Then we searched again with the amino acid sequence of HCA-81/97, NH_2_-ARPPLLGSEEPLCPASR-COOH, not only for the sequences in the coding region of cDNAs but also for the amino acid sequences deduced from sense or antisense nucleotide sequences of cDNAs. This search identified the following three genes: Family with sequence similarity 119, member A (FAM119A), golgi SNAP receptor complex member 1 (GOSR1) and Bardet-Biedl syndrome 5 (BBS5) (Figure 6). It is of considerable interest that the conserved sequences appeared repeatedly as observed in HCA25a. However, all of these repeated sequences were located in the 3'-untranslated region, and some were found in the antisense strands (in the opposite direction). It remains unknown whether these conserved sequences can be translated under certain conditions.

### Expression of ECSA in esophageal SCC

**Figure 7:**
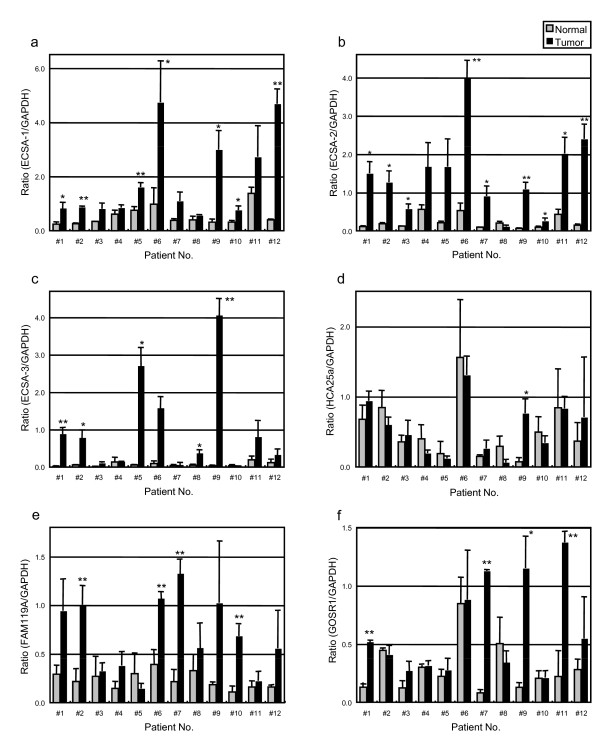
**Expression of ECSA family mRNAs in normal and esophageal SCC tissues**. The expressions of ECSA-1 (a), ECSA-2 (b), ECSA-3 (c), HCA25a (d), FAM119A (e) and GOSR1 (f) mRNA were examined by RT-PCR in specimens of normal and esophageal SCC tissues resected from patients 1 to 12 (#1 - #12). Each value was normalized with that of GAPDH, and the averages of three experiments are shown. The error bars represent SD (n = 3, t test). Asterisks indicate a significant increase in carcinoma tissues as compared to normal tissues. (*, p < 0.05; **, p < 0.01).

The expression of the ECSA family members was examined by realtime RT-PCR, and the results showed that the mRNA expression levels of ECSA-1, -2 and -3 and FAM119A were elevated more frequently in tumor tissues than in the surrounding normal tissues (Figure 7a, b, c and 7e; Additional File 3). The elevation was most frequently seen for ECSA-2 (9 of 12 cases) and was most prominent for ECSA-3 (10 to 40-fold increase in tumor tissues). On the other hand, the expression levels of HCA25a and GOSR1 were relatively low and not apparently different between the tumor and normal tissues (Figure 7d and 7f). The expression of BBS5 was almost undetectable in all specimens (data not shown). Thus, it is possible that some, but not all, of the members of the ECSA family are involved in carcinogenesis of esophageal SCC.

### Immunostaining of ECSA in esophageal carcinoma specimens

**Figure 8:**
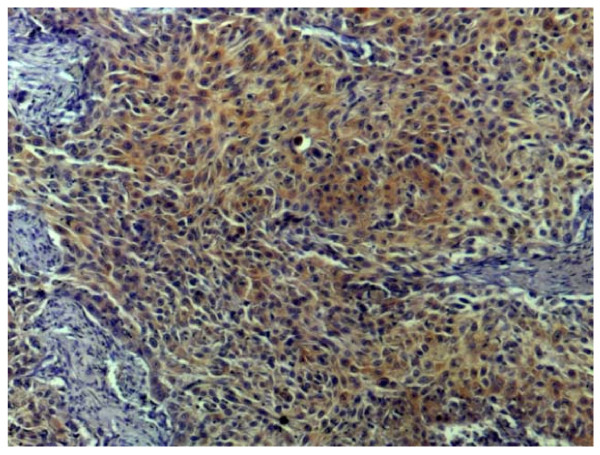
**Immunoperoxidase staining of formalin-fixed paraffin-embedded esophageal carcinoma tissues using pan-ECSA mAb**. Original magnifications, ×100.

We then developed monoclonal antibodies against HCA25a protein and investigated the expression and distribution of ECSA protein by immunohistochemical analysis. Because this monoclonal antibody recognized not only HCA25a but also ECSA-1, -2 and -3 proteins, it was designated as pan-ECSA mAb. We used formalin-fixed esophageal tissue specimens that contained esophageal carcinoma and normal cells of s-ECSA-Abs-positive patients. A representative result (Figure 8) showed that esophageal SCC tissue was more heavily stained than normal esophageal tissue. ECSA proteins appeared to be more abundant in the cytoplasm than in the nucleus.

## Discussion

SEREX screening has been performed in esophageal carcinomas by Chen *et al*. [12] and Tureci *et al*. [26], who successfully identified NY-ESO-I and NY-ESO-II. In the present study, we identified ECSA-1, -2 and -3 as new SEREX antigens of esophageal SCC. Recent studies have shown that serum autoantibodies such as p53 and AFP are useful tumor markers [13-15,27]. s-ECSA-Ab levels examined by ELISA were higher than the cut-off value in 15 - 21% of patients with esophageal SCC (Table 3). Although this rate was not higher than the positive rates of conventional tumor markers such as CYFRA 21-1, CEA and SCC-Ag (24 - 39%) [14], the positive rates of s-ECSA-Abs in healthy donors were less than 2%, indicating very low false-positive rates. Based on the amino acid sequences conserved among ECSA family members, three peptides were synthesized and used for ELISA. One of these peptides, HCA-81/97, was detected with a sensitivity of 22% and a specificity of 100%. Such a high specificity suggests the usefulness of s-ECSA-Abs in the diagnosis of esophageal SCC.

Until we identified three ECSA family members, only one member, HCA25a, had been reported, but with no detailed information. However, three HCA25a-like genes of the chimpanzee have recently been registered in the NCBI database. Thus, the ECSA/HCA25a family may be conserved among primates. In addition to ECSA-1, -2 and -3, three members, FAM119A, GOSR1 and BBS5, were identified by a homology search (Figure 6). We designated this family as the ECSA (esophageal carcinoma SEREX antigen) family but did not include the HCA25a family name because HCA25a was not apparently related to esophageal SCC (Figure 7).

The mRNA expression levels of ECSA-1, -2 and -3 and FAM119A in tumor tissues were frequently higher than those in normal tissues (Figure 7). Consistently, immunohistochemical analysis using a pan-ECSA mAb showed that the expression levels of ECSA proteins were low in normal tissues but high in SCC (Figure 8). Such tumor-specific expression suggests that these ECSA family members may play a crucial role in carcinogenesis. However, none of the isolated ECSA clones (ECSA-1, -2 and -3) contained apparent initiation codons (Additional File 1), and the conserved sequence was identified frequently in the 3'-untranslated regions of the family members (Figure 6). It is plausible that they are expressed by alternative splicing under certain conditions in tumor cells. The development of serum autoantibodies against the conserved sequence of the ECSA family suggests that the conserved domain in some members of the ECSA family was translated into proteins. Nevertheless, it cannot be ruled out that ECSA RNA may not exert an effect through its peptide product but instead may act as antisense RNA or microRNA molecules [28]. Alternatively, the DNA region harboring the conserved ECSA sequence may be a binding site of chromatin modifiers such as histone acetyltransferase, and thereby, enhance gene expression.

TROP-2 [16], SURF1 [17], SLC2A1 [18], TRIM21 [19], myomegalin [21], UBE2I [22], AISEC [23], CUEC-23 [24] and makorin1 [25] are novel tumor marker SEREX antigens of esophageal SCC. Among these markers, ECSA has shown the highest specificity. Although the presence of s-ECSA-Abs was partly associated with a poor prognosis (Figure 4), further trial data might verify the validity of ECSA as a diagnostic marker.

We evaluated the clinical significance of s-ECSA-Abs in patients with esophageal SCC. The presence of s-ECSA-Abs was partly associated with a poor prognosis (Figure 4). The very low false-positive reactivity of s-ECSA-Abs (Table 2) suggests that they are useful as new diagnostic markers for esophageal SCC not only by themselves but also in combination with other conventional tumor markers.

## Conclusions

We identified ECSA-1, -2 and -3 as novel esophageal SCC SEREX antigens, and designated them as the ECSA family including FAM119A. Serum antibodies against ECSA proteins or a synthetic peptide with a conserved amino acid sequence among the ECSA family members showed a sensitivity higher than 15% and a specificity higher than 98% for patients with esophageal SCC. These results suggests that they are useful as new diagnostic markers for esophageal SCC.

## Methods

### Human esophageal squamous cell carcinoma cDNA libraries

Sera were collected from patients after they had given written informed consent. This study was approved by the Local Ethical Review Board of the Chiba University, Graduate School of Medicine. Recombinant DNA work was performed with official permission of the Chiba University, Graduate School of Medicine, in accordance with the rules of the government of Japan. The human esophageal SCC cell line, T.Tn [29,30], was established by the Department of Clinical Molecular Biology, Chiba University, Graduate School of Medicine. Total RNA was prepared from T.Tn cells by the acid guanidine thiocyanate-phenol-chloroform method [31] and purified by binding to poly(A)^+^RNA using Oligotex-dT_30 _(Super) mRNA Purification Kit (Takara Biochemicals, Kyoto, Japan) according to the manufacturer's instructions. cDNA was ligated into the EcoRI-XhoI site of the λZAP II phage. The original library size was 1.8 × 10^6^.

### Patients and healthy donor sera

Sera were obtained from 146 patients with esophageal SCC and 118 healthy donors. The patients with esophageal SCC included 129 men (89%) and 17 women (11%), with a median age of 65 years (range 44 to 82 years). Before starting treatment, the 146 cancer patients were classified according to the Tumor Node Metastasis/Union Internationale Contre le Cancer (TNM/UICC) classification system [32] as follows: stage I (n = 32), stage II (n = 29), stage III (n = 42), and stage IV (n = 43). Of the SCC patients, 57 underwent R0 resection with extended lymph node dissection and 89 received definitive chemo-radiation therapy. After treatment, they were followed with clinical examinations and imaging studies on a regular basis until death or until the end of March 2007, whichever occurred first. The mean follow-up time for survivors was 30 months. Each serum sample was centrifuged at 3,000 × g for 5 min, and then the supernatant was stored at -80°C until use. Repeated thawing and freezing of samples were avoided.

### Immunological screening of the esophageal carcinoma cell antigens by SEREX

Esophageal carcinoma antigens were screened by the SEREX method previously published by Sahin *et al*. [10]*E. coli *XL1-Blue MRF' was infected with λZAP II phages containing the cDNA library, and the expression of cDNA was induced by blotting on nitrocellulose membranes (NitroBind, Osmonics Inc., Minnetonka, MN) that had been pretreated for 30 min with 10 mM isopropyl β-D-thiogalactoside (IPTG; Wako Pure Chemicals, Osaka, Japan). The membranes were then washed three times with TBS-T [20 mM Tris-HCl (pH 7.5), 0.15 M NaCl and 0.05% Tween-20], and blocking was performed by treatment with 1% protease-free bovine serum albumin (Wako Pure Chemicals) in TBS-T for 1 h. The membranes were exposed in 1:2,000-diluted serum with no preabsorption for 1 h. After washing with TBS-T three times, the membranes were treated for 1 h with 1:5,000-diluted alkaline phosphatase-conjugated Fc fragment-specific goat anti-human IgG (Jackson ImmunoResearch Laboratories, West Grove, PA). Positive reactions were detected by incubation in color development solution (100 mM Tris-HCl (pH 9.5), 100 mM NaCl and 5 mM MgCl_2_) containing 0.3 mg/ml of nitroblue tetrazolium chloride (Wako Pure Chemicals) and 0.15 mg/ml of 5-bromo-4-chloro-3-indolyl-phosphate (Wako Pure Chemicals). Positive clones were recloned twice to obtain monoclonality and retested for serum reactivity.

### Sequence analysis of identified antigens

Monoclonalized phage cDNA clones were converted to pBluescript phagemids by *in vivo *excision using ExAssist helper phage (Stratagene, La Jolla, CA). Plasmid DNA was obtained from an *E. coli *SOLR strain transformed by the phagemid. The cDNA inserts were sequenced by the dideoxy chain termination method using the DNA Sequencing kit BigDye™ Terminator (Applied Biosystems, Foster City, CA) and ABI PRISM 3700 DNA Analyzer (Applied Biosystems). Sequences were analyzed for homology with public databases of known genes and proteins using NCBI-BLAST.

### Western blotting analysis

A total of 40 patients were analyzed by Western blot analysis. *E. coli *JM109 cells, which contained cDNA clones recombined in pBluescript II, were cultured with or without 1 mM IPTG for 2.5 h. Cells were then washed with phosphate-buffered saline (PBS) and lysed by incubation at 100°C for 3 min in SDS sample buffer [33]. *E.coli *lysate was then subjected to SDS-PAGE followed by Western blotting using sera of patients or healthy donors as described previously [16,18,19].

### Purification of recombinant ECSA proteins

The cDNA insert of ECSA incorporated in pBluescript was cleaved by EcoRI and XhoI, and recombined in pGEX-4T-3 (Amersham Biosciences, Piscataway, NJ). *E.coli *JM109 cells containing pGEX-4T-3-ECSA or control pGEX-4T-3 were cultured in 200 ml of Luria broth (LB) and treated with 0.1 mM IPTG for 2.5 h. Cells were harvested, washed with PBS and lysed by sonication in 10% Trinton X-100, 50 mM Tris-HCl (pH 8.0), 1 mM ethylenediaminetetraacetic acid (EDTA) and 1 mM dithiothreitol (DTT). The lysate was then centrifuged at 10,000 × g for 30 min at 4°C. Glutathione-S-transferase (GST) in the supernatant was directly purified by glutathione-Sepharose (Amersham Biosciences). The purified proteins were concentrated by Apollo centrifugal concentrators (Orbital Biosciences, Topsfield, MA).

### Enzyme-linked immunosorbent assay (ELISA)

A total of 72 patients were analyzed by ELISA. Fifty μl of antigens (GST or GST-ECSA) diluted to 10 μg protein/ml in PBS were added to the wells of a microtiter plate, and incubated at room temperature overnight. The plate was washed four times with 0.1% Tween-20 in PBS (PBS-T) and then blocked with 10% fetal bovine serum in PBS (PBS-FBS). The plate was incubated at room temperature for 1 h and washed four times with PBS-T. Fifty μl of the respective sera diluted 1/100 in PBS-FBS were added to the wells and incubated for 1 h. The wells were washed with PBS-T four times, and the bound IgG antibodies were detected by incubation with horseradish peroxidase-conjugated anti-human IgG antibody (Jackson ImmunoResearch Laboratories) for 1 h, followed by washing and the addition of 100 μl of a peroxidase substrate (*o*-phenylenediamine, 0.4 mg/ml) in citrate-phosphate buffer, pH 5.0, containing 0.02% (v/v) H_2_O_2_. The reaction was stopped with 30 μl of 4 M H_2_SO_4_. The absorbance at 490 nm was determined with a microplate reader (Emax, Molecular Devices, Sunnyvale, CA).

For sandwich ELISA, micro-titer plates were coated overnight with 50 μl of 10 μg/ml of streptavidin (Wako Pure Chemicals) in 0.05 M carbonate/bicarbonate buffer (pH 9.6). Plates were blocked with 150 μl of PBS-FBS for 1 h, washed four times with PBS-T and incubated overnight with 50 μl of purified biotinylated synthetic peptides (10 μg/ml) in PBS-T. The plate was then washed four times with PBS-T, and 50 μl of serum diluted 1/100 in PBS-FBS were added to each well.

### Reverse transcription-PCR (RT-PCR)

Total cellular RNA was isolated from the tumor tissues using FastPure RNA Kit (Takara Bio). Reverse transcription was carried out with oligo(dT)_20 _primer using the ThemoScript RT-PCR System (Invitrogen, Carlsbad, CA), according to the manufacturer's instructions. The presence of ECSA transcripts was established via quantitative realtime RT-PCR using the following primers and probes (Universal Probe Library; Roche, Basel, Switzerland).

ECSA-1 sense: 5'-TAAGGGCGGTGCAAGATG-3'

ECSA-1 antisense: 5'-TAGGGAGTGGTGCTGACTCTTA-3'

ECSA-1 probe: #8

ECSA-2 sense: 5'-GAGACTTTTCATTTTGTTCTGCACT-3'

ECSA-2 antisense: 5'-GGGGGTAAGGTCACAGATCA-3'

ECSA-2 probe: #6

ECSA-3 sense: 5'-GACATGGGAGACTTTTCATTTTG-3'

ECSA-3 antisense: 5'-CTGTGGGATTGGTGGTGATA-3'

ECSA-3 probe: #29

FAM119A sense: 5'-AATGCAGTTCTTTCCCAAGC-3'

FAM119A antisense: 5'-TTTTAATGTCATCCTATGCGTTG-3'

FAM119A probe: #38

GOSR1 sense: 5'-TGACGTTGGACGACAAAGAT-3'

GOSR1 antisense: 5'-GTCGAGCCTGTTTCCTGAGA-3'

GOSR1 probe: #48

BBS5 sense: 5'-CAGTCCCATATTTGGAGTTGATT-3'

BBS5 antisense: 5'-TCGACTGTCAGAGCTTCGAG-3'

BBS5 probe: #45

HCA25a sense: 5'-CGTTCTTGAAAATCTGTTCCTCTT-3'

HCA25a antisense: 5'-AGAGGGAGACCGTGGAAAG-3'

HCA25a probe: #52

hGAPDH sense: 5'-AGCCACATCGCTCAGACA-3'

hGAPDH antisense: 5'-GCCCAATACGACCAAATCC-3'

hGAPDH probe: #60

PCR was performed using Fast Start Taq Man probe Master (Roche) and ABI PRISM 7000 Sequence Detection System (Applied Biosystems LLC) as follows: an initial denaturation step at 95°C for 2 min, followed by 55 cycles of denaturation at 95°C for 15 sec and annealing/extension at 60°C for 30 sec. The -ΔΔC_T _mean and SD were calculated and normalized to the GAPDH value.

The presence of ECSA transcripts was also examined by standard PCR using the following primer pairs: ECSA-3 sense, 5'-GACATGGGAGACTTTTCATTTTG-3'; ECSA-3 antisense, 5'-CTGTGGGATTGGTGGTGATA-3'; β-actin sense, 5'-GGATCAGCAAGCAGGAGTATG-3'; β-actin antisense, 5'-GAGAAGTGGGGTGGCTTTTAG-3'. PCR was performed using KOD-Plus-DNA polymerase (Toyobo, Osaka, Japan) as follows: an initial denaturation step at 94°C for 2 min, followed by 36 cycles for ECSA-3, or 28 cycles for β-actin, of denaturation at 94°C for 15 sec, annealing at 60°C for 30 sec, and extension at 68°C for 30 sec, with a final extension of 5 min at 68°C using Takara PCR Thermal Cycler SP (Takara Bio, Otsu, Japan).

### Preparation of HCA25a antibody

Monoclonal anti-HCA25a antibody was developed by immunizing mice with recombinant HCA25a protein. The antibody obtained from the culture medium of the hybridomas reacted with ECSA-1, -2 and -3, and was designated as pan-ECSA mAb.

### Immunohistochemical staining for HCA25a protein

Paraffin-embedded esophageal SCC tumor tissues were cut and dewaxed through alcohol/xylene gradient solutions. After antigen retrieval by incubation at 95°C for 40 min in 10 mM citrate buffer (pH 6.0), endogenous peroxidase was blocked with 3% hydrogen peroxide in methanol for 15 min. After blocking with 5% non-fat dry milk in PBS for 30 min, sections were incubated for 1 h with pan-ECSA mAb diluted at 1:200. Parallel sections were incubated with 5% non-fat dry milk as negative controls. All sections were washed three times with wash buffer (DAKO Corporation, CA) for 5 min each. For linking, all sections were incubated with biotinylated anti-mouse IgG, and reacted with streptavidin-conjugated horseradish peroxidase reagent (DAKO LSAB^®+^HRP system). Finally, the reaction was visualized with a chromogen, diaminobenzidine, in 3% hydrogen peroxidase. Sections were then counterstained with hematoxylin, dehydrated and mounted.

### Statistical Analyses

Fisher's exact (two-sided) probability test and the Mann-Whitney U test were used to determine the significance of the differences between two groups. The survival probabilities were calculated using the product-limit method of Kaplan and Meier, considering all deaths. Survival differences between groups were determined using the log-rank test. All statistical analyses were carried out using the Stat View 5.0J program for Windows (SAS Institute Inc., Cary, NC). P values lower than 0.05 were considered statistically significant.

## List of abbreviations used

EDTA: ethylenediaminetetraacetic acid; ELISA: enzyme-linked immunosorbent assay; FBS: fetal bovine serum; GST: glutathione-S-transferase; IPTG: isopropyl β-D-thiogalactoside; PBS: phosphate-buffered saline; RT-PCR: reverse transcription-polymerase chain reaction; SCC: squamous cell carcinoma; SEREX: serological identification of antigens by recombinant cDNA expression cloning; s-ECSA-Abs: serum-ECSA-antibodies.

## Competing interests

Takaki Hiwasa and Hideaki Shimada have received research support from Medical & Biological Laboratories Co., Ltd. in 2005 - 2007.

## Authors' contributions

THi, HS, and MT designed the overall research plan. AK, TS, MK, KNF, and MY performed SEREX screening and carried out ELISA, SK, THa, and AM prepared antigen proteins, YT designed the antigen peptides, and FN, TO, TN, HM prepared tissue samples and carried out expression analysis. All authors read and approved the final manuscript.

## Supplementary Material

Additional file 1 **Nucleotide and amino acid sequences of ECSA-1, -2 and -3**. Coding regions of the nucleotide sequences are underlined. Click here for file

Additional file 2 **Purification of recombinant ECSA-2 protein using glutathione-Sepharose**. cDNA of ECSA-2 was recombined into pGEX-4T, which produced the GST-ECSA-2 fusion protein after treatment with IPTG. The fusion protein was affinity-purified by glutathione-Sepharose. 1, total extract; 2, supernatant fraction after lysis with Triton X-100 followed by centrifugation; 3: precipitate fraction after lysis with Triton X-100 followed by centrifugation; 4: flow-through/unbound fraction, 5: glutathione-eluted fraction. Coomassie-stained SDS-polyacrylamide gel is shown. Click here for file

Additional file 3 **Expression of ECSA-3 mRNA in normal and esophageal SCC tissues**. The expressions of ECSA-3 and β-actin (lower panel) mRNA were examined by RT-PCR in specimens of normal (N) and carcinoma (T) tissues resected from patients 1 to 7 (P1 - P7). N* and T* represent the products from RNA of normal esophageal keratinocytes obtained from Cybrdi and the T.Tn esophageal SCC cell line, respectively. Click here for file
