# Supplementary material for: Identification of a novel SEREX antigen family, ECSA, in esophageal squamous cell carcinoma
Source: Proteome Sci. 2011 Jun 23;9:31. doi: 10.1186/1477-5956-9-31 (PMC3135497; doi:10.1186/1477-5956-9-31)
Supplement: Additional file 1 — Nucleotide and amino acid sequences of ECSA-1, -2 and -3. Coding regions of the nucleotide sequences are underlined. [file 1477-5956-9-31-S1.DOC]

**Additional File 1**

Nucleotide sequence of ECSA-1

GGCCAGCTGCCCCATCTGGGAGGGAGGTGGGGGGGTCAGCCCCCCGCCCGGCCAGCCGCCCCGTCCGGGAGGTGAGGGGCGCCTCTGCCCGGGCGCCCCTACTGGGAAGTGAGGAGCCACTCTGCCCGGCCAGCCGCCCCGTCCGGGAGGGAGGTGGCGGGGTCAGCCCCCCACCTGGCCAGCCGCCCCGTCCGGGAAGTGAGGGGCGCCTCTGCCCGGCTGCCCCTACTGGGAAGTGAGGAGCCACTCTGCCCGGCCAGCCGCCCCGTCCGGGAGGGAGGTGGGGGGGTCAGCCCCCCGCCCGGCCAGCCGCCCCGTCCGGGAGGTGAGGGGCGCCTCTGCCCGGCCGCCCCTACTGGGAAGTGAGGAGCCCCTCTGCCCGGCCAGCCGCCCCGTCCAGGAGGGAGGTGGGGGGGGTCAGCCCCCCGTCCGGCCAGCCGCCCCGTCCGGGAGGTGAGGGGCGCCTCTGCCCGGCGGCACCTACTGGGAAGTGAGGAGCCCCTCTGCCCGGCCACCACCCCGTCTGGGAGGTGTACCCAACAGCTCATTGAGAACGGGCCATGATGACAATGGCGGTTTTGTAGAATAGAAAGGGGGGAAAGGTGGGGAAAAGATTGAGAAATCGGATGGTTGCCGTGACTGTGTGGAAAGAGGTAGACATGGGAGACTTTTCATTTTGTTCTGTACTAAGAAAAATTCTTCTGCCTTGGGATCCTGTTGATCGGTGACCTTACCCCCAACCCGGTGCTCTCTGAAACATGTGCTGTATCCACTCAGGGTTGAATGGATTAAGGGCGGTGCAAGATGTGCTTTGTTAAACAGATGCTTGAAGGCAGCATGCTCCTTAAGAGTCAGCACCACTCCCTAATCTCAAGTACCCAGGGACACAAACACTGCGGAAGGCGGCAGGGTCCTCTGCCTAGGAAAACCAGAGACCTTTGTTCACTTGTTTATCTGCTGACCTTCCCTCCACTACTGTCCTGTGACCCTGCCAAATCCCCCTCTGCGAGAAACACCCAAGAATGATCAATAAAAAATAATAATAATAATAATAAAATTAAATTAAATTAAAAAAAAATTGCCCAAAGGATTTTTCAAAGGAAAAATTAAGATTTGGGTCAGATTCTATCCTTGGCTATATGTGTGAGTTCAATACTAAAAGCAACATGGAAATCTGACCTTTCCCAAGGAACTGTTTCACCTAAGAACCTACAAGAAATTCGGATTTGATTAACAGCTCAGAGCTCATCTCTTCCACCTGACCACTTCCTACTTCTTGGTTTATAAGTACGAGGTGCTTAGGAAGAACGATGTGTTCAAGAATGGGAAAATTATCCCTACTGGCCCCCCCAGCGCAACCTTGTGCTGCACACCCTGACAGAAAGAATAGAGAAGTCGTTTACTTATTACAGTGAGAGGTGGCTTCAATCATCATGTTGACCTGACCTGCAAAGGAAGGCTTGTAATTCATAATTAAACAGAGCACCACTGCCTGCCTTTTCCAGATAAAATTACATTTTGAGAGAGAAAAAAATTATACTCATTTTGATTTCACAACTTTAAACAATGGAATGTTGGGAAACTATTCTTGACAAAGCAGCCTGCTATACATTCACCCAACCACCTGTCACTAACATCATTCTCAAACACAAAGGGCTATTTTTATGTAGTACATTACCAAGATAGATAATATCCCATTGCCATAGCAACAAGTTTTCCAGAAGATATGTTAACCCATGAAGAATGCAAAACAGAACAAAGCTATTTCTTTGCATTTGCCTTTAAAATATATGAAGAGGATTGTAAGATTTTTAAGGCGTGGTTTGCTCAATAGAGCCTTAGTGGATATGAGCAATGTATAAAGAAATCCTTAGGCCATTGACCACTGCCTA

Nucleotide sequence of ECSA-2

GGCCGGCCGCCCCTACTGGGAAGTGAGGAGCCCCTCTGCCCGGCCAGCCGCCCCATCCGGGAGGGAGGTGGGGGGGGTCAGCCCCCTGCCCGGCCAGCCACCCTGTCTGGGAGGGAGGTGGGGGGGTCAGCCCTCCGCCCGGCCAGCCGCCCCGTCTGGGAGGTGAGGGGCGCCTCTGCCCGGCCGCCCCTACTGGGAAGTGAGGAGCCCCTCTGCCCGGCCAGCCGCCCTGTCTGGGAGGGAGGTGGGGGGGTCGGCCCCCCGCCCGGCCAGCCGCCCCATCCGGGAGGGAGGTGGGGGGGTCGGCCCCCATCCCGGCCAGCCGCCCCGTCCGGGAGGGAGGTGGGGGGGGTCAGCCCCCACGCCCGGCCAGCCGCCCCGTCCGGGAGGTAAGGGGCGCCTCTGCCCGGCCGCCCCTACTGGGAAGTGAGGAGCCCCTCTGCCCAGCCAGCCGCCCTGTCCGGGAGGGAGGTGGGGGGTTCAGCCCCCCGCCCGGCCAGCCGCCCCGTCCGGGAGGGAGGTGGGGGGGGGGGTCAGCCCCCCTGCCCGGCCAGCCGCCCCGTCCGGGAGGTGAGGGGCGCCTCTGCCCGGCCGCCCCTACTGGGAAGTGAGGAGCCCCTCTGCCCGGCCACCACCCCGTCTGGGAGGTGTGCCCAATAGCTCATTGAGAACGGGCCAGGATGACAATGGCGGCTTTGTGGAATAGAAAGGCGGGAAAGGTGGGGAAAAGATTGAGAGATCGGATGGTTGCCGTGTCTGTGTGGAAGGAAGTAGACATGGGAGACTTTTCATTTTGTTCTGCACTAAGAAAAATTCCTCTGCCTTGGGATCCTGTTGATCTGTGACCTTACCCCCAACCCTGTGCTCTCTGAAACATGTGCTGTGTCCACTCAGAGTTAAATGGATTAAGGGCGGTGCAAGATGTGCTTTGTTAAACAGATGCTTGAAGGCAGCATGCTCGTTAAGAGTCATCACCACTCCCTAATCTCAAGTACCCAGGGACACAAACGCTGCGGAAGGCCGCAGGGTCCTCTGCCTAGGAAAACCAGAGACCTTTGTTCACTTGTTTATCTGCTGACCTTCCCTCCACTATTGTCCCATGACCCTGCCAAATCCCCCTCTGTGAGAAACACCCAAGAAT

Nucleotide sequence of ECSA-3

AACCTGAGCTCCACCGCGGTGGCGGCCGCTCTAGAACTAGTGGATCCCCCGGGCTGCAGGAATTCGGCACGAGGGCCAGCCGCCCCGTCCGGGAGGGAGGTGGGGGGATCAGCCCCCAGCCTGGCCAGCCGCTCTGTCCTGGAGGTGAGGGGCGCCTCTTCCCGGCCGCCCCTACTGGGAAGTGAGGAGCCCCTCTGCCCGGCCAGCCGCCCCGTCCGGGAGGGAGGCGGGGGGGGGGGTCTCT

Putative partial amino acid sequence of ECSA-1

PAAPSGREVGGSAPRPASRPVREVRGASARAPLLGSEEPLCPASRPVREGGGGVSPPPGQPPRPGSEGRLCPAAPTGK

Putative partial amino acid sequence of ECSA-2

GRPPLLGSEEPLCPASRPIREGGGGGQPPARPATLSGREVGGSALRPASRPVWEVRGASARPPLLGSEEPLCPASRPVWEGGGGVGPPPGQPPHPGGRWGGRPPSRPAAPSGREVGGVSPHARPAAPSGR

Putative partial amino acid sequence of ECSA-3

NLSSTAVAAALELVDPPGCRNSARGPAAPSGREVGGSAPSLASRSVLEVRGASSRPPLLGSEEPLCPASRPVREGGGGGGL
