# Supplementary figures and images for: Identification of a novel SEREX antigen family, ECSA, in esophageal squamous cell carcinoma
Source: Proteome Sci. 2011 Jun 23;9:31. doi: 10.1186/1477-5956-9-31 (PMC3135497; doi:10.1186/1477-5956-9-31)

## Slide 1
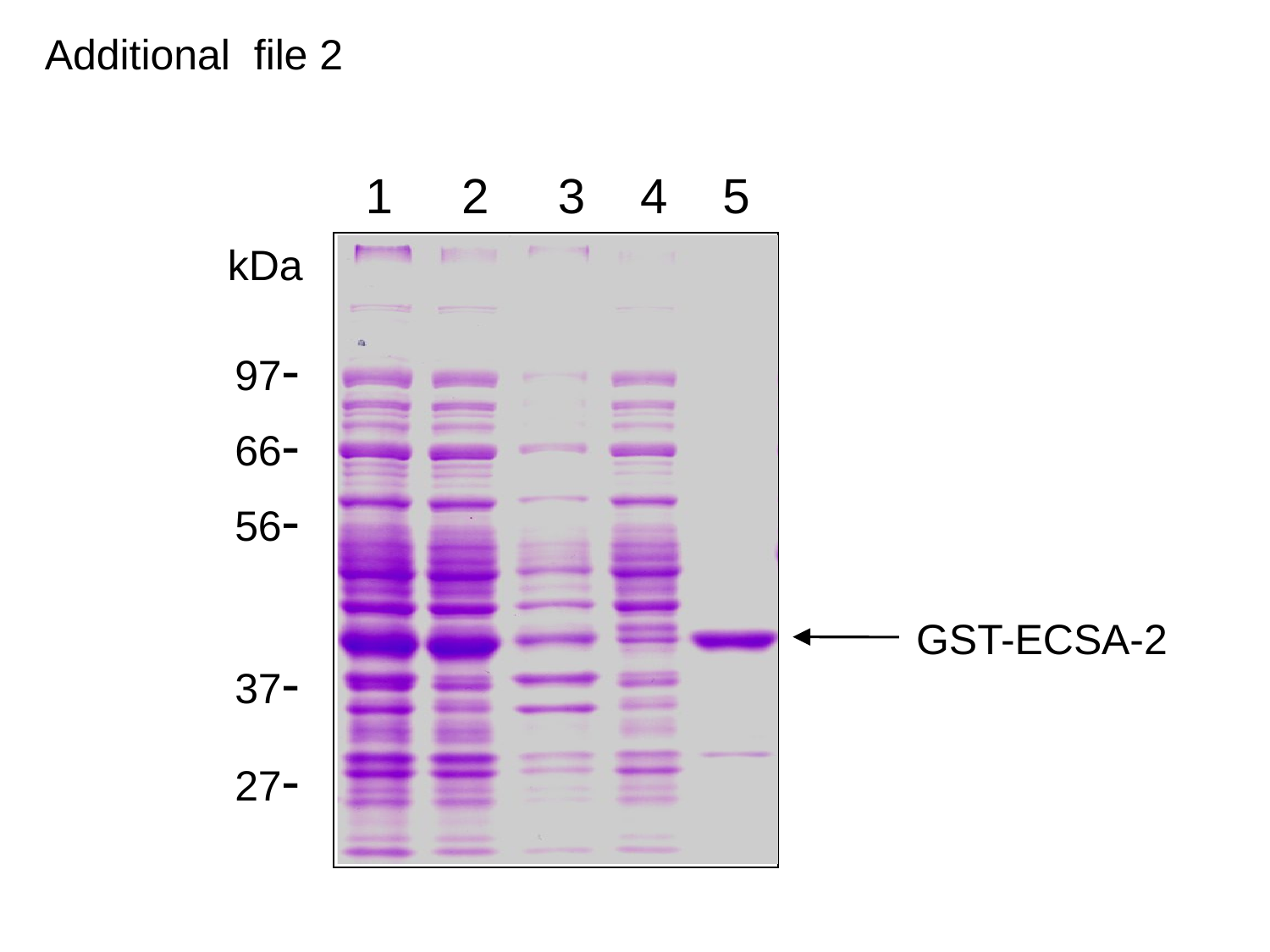

Additional file 2
 1 2 3 4 5
97-
66-
56-
GST-ECSA-2
37-
27-
kDa

Supplement: Additional file 2 — Purification of recombinant ECSA-2 protein using glutathione-Sepharose. cDNA of ECSA-2 was recombined into pGEX-4T, which produced the GST-ECSA-2 fusion protein after treatment with IPTG. The fusion protein was affinity-purified by glutathione-Sepharose. 1, total extract; 2, supernatant fraction after lysis with Triton X-100 followed by centrifugation; 3: precipitate fraction after lysis with Triton X-100 followed by centrifugation; 4: flow-through/unbound fraction, 5: glutathione-eluted fraction. Coomassie-stained SDS-polyacrylamide gel is shown. [file 1477-5956-9-31-S2.PPT]

## Slide 1
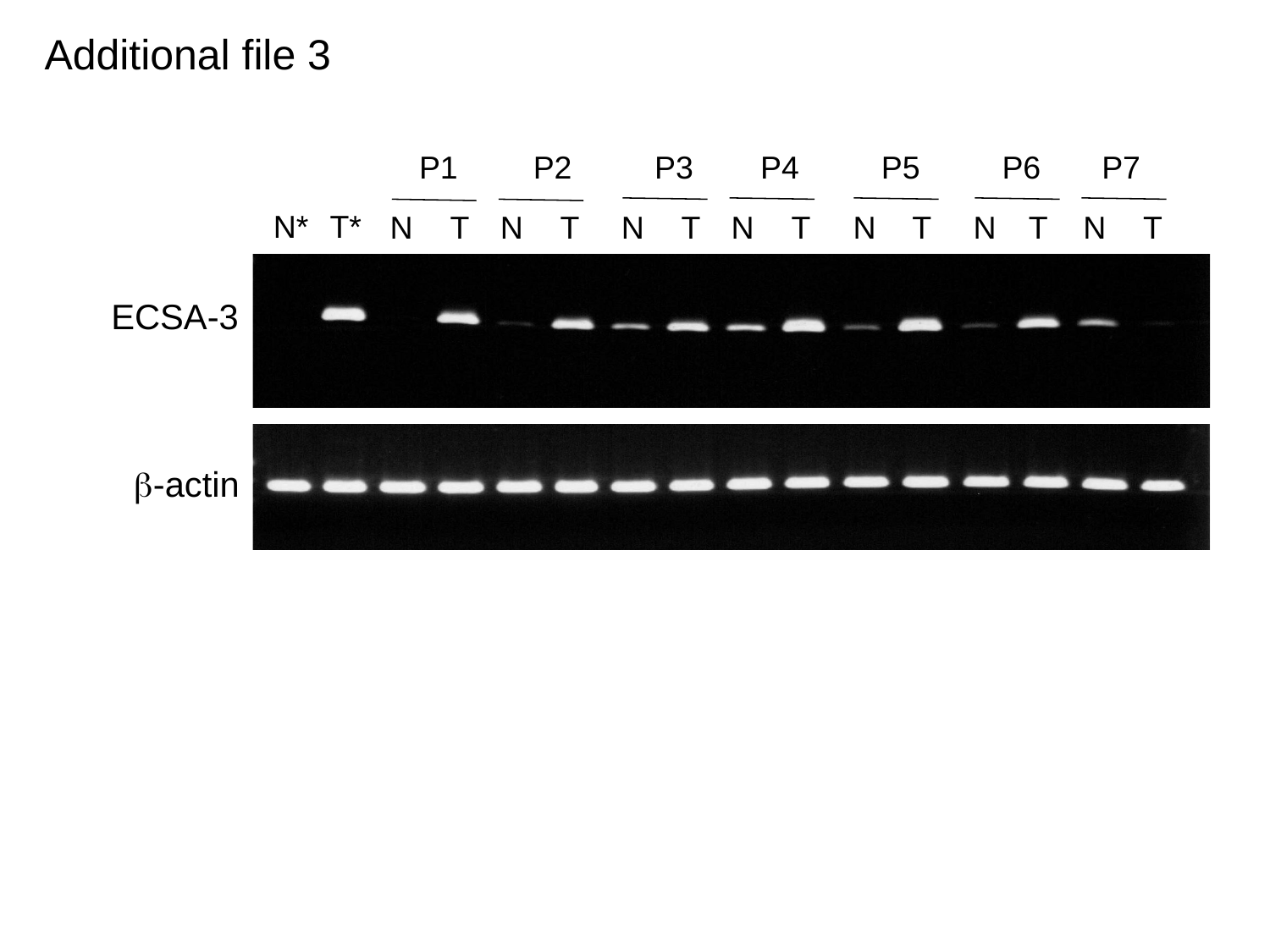

Additional file 3
P5
P1
P2
P3
P4
P6
P7
N*
T*
N
T
N
T
N
T
N
T
N
T
N
T
N
T
ECSA-3
 β-actin

Supplement: Additional file 3 — Expression of ECSA-3 mRNA in normal and esophageal SCC tissues. The expressions of ECSA-3 and β-actin (lower panel) mRNA were examined by RT-PCR in specimens of normal (N) and carcinoma (T) tissues resected from patients 1 to 7 (P1 - P7). N* and T* represent the products from RNA of normal esophageal keratinocytes obtained from Cybrdi and the T.Tn esophageal SCC cell line, respectively. [file 1477-5956-9-31-S3.PPT]
